# The effect of HPV vaccination on the rate of high-grade cytology in 25-year-old women attending cervical screening in Ireland

**DOI:** 10.1007/s11845-023-03551-y

**Published:** 2023-10-19

**Authors:** Micheal Rourke, Patricia Fitzpatrick, Olalekan Popoola, Rewhandamzi Boms, Therese Mooney, Laura Heavey, Caroline Mason Mohan, Cara M. Martin, Lucy Jessop, Noirin E. Russell

**Affiliations:** 1 National Screening Service, Kings Inns House, Parnell St, Dublin 1, Dublin, Ireland; 2 School of Public Health, Physiotherapy & Sports Science, UCD, Dublin, Ireland; 3 School of Medicine Trinity College Dublin, Dublin, Ireland; 4 Trinity St James Cancer Institute, Trinity College Dublin, Dublin, Ireland; 5 National Immunisation Office, Dublin, Ireland; 6 School of Medicine, University College Cork, Cork, Ireland

**Keywords:** High-grade CIN, HPV vaccination

## Abstract

**Introduction:**

Women vaccinated through the initial catch-up HPV vaccination programme (2011/12 to 2013/14) first became eligible for cervical screening in 2019 at age 25. This study aims to examine the changes in detection of HG cytology outcomes in 25-year-olds screened from 2010 to 2022 compared to population data on HPV vaccination in this group.

**Methods:**

This was an ecological-type study. Cytology results from the CervicalCheck database from 2010 to 2022 (High Grade, Low Grade, and No Abnormality Detected) were plotted against data from the National Immunisation Office on the uptake of HPV vaccinations in females from 2010 to 2022.

**Results:**

Vaccination rates in the catch-up programme were lower (44–70%) than for routine HPV immunisation at age 12/13 in 2010/11 (81%). The rate of high-grade cytology in 25-year-olds in 2015–2018 was 3.7% of all cytology tests taken in this age group. For the corresponding period from 2019 to 2022 (when vaccinated women were attending screening), the average percentage of HG cytology in 25-year-olds was 1.5%, representing a significant reduction in HG cytology proportions (*p* < 0.001).

**Conclusion:**

This study provides early evidence of the potential impact of HPV vaccination on cervical disease in the Republic of Ireland. Despite lower vaccination uptake in the initial catch-up group, we are seeing early signs of the positive protective effect of HPV vaccination in women at the time of their first cervical screening test. Plans to incorporate individual-level HPV vaccination status for women on the cervical screening register will allow more detailed assessment of the impact of HPV vaccination.

## Introduction

Cervical cancer, though preventable, remains one of the most common cancers and causes of cancer-related mortality among women globally [1]. Approximately 70% of cervical cancers are caused by persistent infection with Human Papilloma Virus (HPV) subtypes 16 and 18 [2]. Vaccination targeting these 2 subtypes has been available in Ireland since 2010/11. The vaccine is most effective when administered prior to sexual debut, in a HPV-naïve population [3]. The vaccination programme was rolled out through schools, starting in 2010 in those aged 12–13 years in first year of secondary school and age equivalent in non-secondary schools (special schools and home schools). A 3-year catch-up vaccination campaign subsequently commenced in 2011/2012 for girls in sixth year of secondary school and their age equivalent in non-secondary schools [4] (aged 17/18 years) [1]. The Gardasil 4 vaccine was used initially, with three doses required.

Initial vaccination uptake was good for first years and lower for sixth-year students. However, the proportion of girls who completed the vaccination schedule in 2015–2016 dropped to 72.3%, and uptake of the first dose decreased further across all areas to an estimated 50% in 2016–2017. Despite the European Medicines Agency stating there were no safety concerns with the HPV vaccine, inaccurate information was spread by lobby groups using emotive personal narratives on social media platforms [5]. By prioritising content that elicits strong emotional responses rather than nuanced content, social media accelerates the spread of false information and widens its audience [6]. However, a concerted effort from the HPV Vaccination Alliance, bringing together 38 organisations to campaign positively about HPV vaccination, resulted in an increase in vaccination rates in the following years [5]. This campaign was helped by the tireless campaigning of the patient advocate Laura Brennan, a young woman who died at the age of 26 from cervical cancer [5].

The Irish cervical screening programme, CervicalCheck, commenced in 2008 as a cytology-based programme that provided almost 3.2 million screening tests, achieved coverage of about 78.7%, and helped reduce the incidence of cervical cancer in Ireland by 2.8% per year in its first 12 years [7]. During this period screening was provided every 3 (for those aged 25–45 years) to 5 years (for those aged 45–60 years, after 2 normal screens). In March 2020, on the recommendation of the Health Information and Quality Authority, the programme transitioned to primary HPV screening with reflex cytology tests for HPV-positive cases [7]. In comparison to over 90% for HPV testing, cytology has a sensitivity of about 50 to 70% for identifying precancer [8].

To reduce the global burden of cervical cancer-related morbidity and mortality, the World Health Organisation (WHO) in 2018 announced a global call to action to eliminate cervical cancer (achieve an incidence threshold of 4 per 100,000 women-years) which was adopted in 2020 [2]. The elimination goal (known as the 90-70-90 plan) is based on 3 key pillars: achieving and maintaining high coverage targets for HPV vaccination (90% of girls fully vaccinated with HPV vaccine by age 15 years), screening (70% of women are screened with a high-performance test by 35 years of age and again by 45 years of age) and treatment of precancerous lesions, and management of cancer (90% of women with precancer and invasive cancer treated and managed) by 2030 and beyond [2].

International evidence strongly suggests a reduction in the rate of high-grade (HG) cytology in HPV-vaccinated populations [9]. Women vaccinated through the initial catch-up HPV vaccination programme (2011/12, 2012/13 and 2013/14) [4, 10, 11] first became eligible for cervical screening in 2019. This study aims to examine the changes in detection of HG cytology outcomes in 25-year-olds screened from 2010 to 2022 compared to population data on HPV vaccination.

## Methods

This study was ecological in nature. Cytology results from the CervicalCheck database were analysed from 2010 to 2022. Data was obtained from the National Immunisation Office on the uptake of HPV vaccinations in school-going females from 2010 to 2022. The rates of High Grade (HG), Low Grade (LG), and No Abnormality Detected (NAD) cytology were calculated against the baseline of the entire amount of cytology tests performed on 25-year-old females in those same time periods. The data for 2020 was split into two sections to account for the end of primary cytology and the beginning of HPV screening (Mar/April) for descriptive purposes only. Inconclusive/unsatisfactory results were excluded. The percentages of HG cytology detected were overlaid with the vaccination rates in the catch-up programme for HPV vaccination.

A chi-square test was used for comparison of proportions. A linear-by-linear association chi-square test was applied to examine the change in HG cytology between 2015 and 2022. SPSS version 27 was used for analysis.

As this was an ecological study using publicly available data on HPV vaccination and routinely recorded anonymised data from CervicalCheck, ethical review was not sought.

## Results

In Ireland, the girls who were offered HPV vaccination in 6th year of secondary school as part of the initial catch-up campaign would have turned 25 and become eligible for cervical screening in 2019. Table 1 shows national HPV catch-up vaccination rates between 2011/2012 and 2014/2015 and documents the corresponding year in which these women became eligible for screening.

**Table 1:** HPV vaccination rates in 25-year-old women

**Year vaccine administered**	**Vaccination rate (%)**	**Year vaccinated cohort was eligible for cervical screening**
2011/2012 (catch-up programme) [4]	71.5%	2019
2012/2013 (catch-up programme) [10]	67.8%	2020
2013/2014 (catch-up programme) [11]	44.8%	2021
2009/10/11 (normal administration programme)	81.9%	2022

**Fig. 1:**
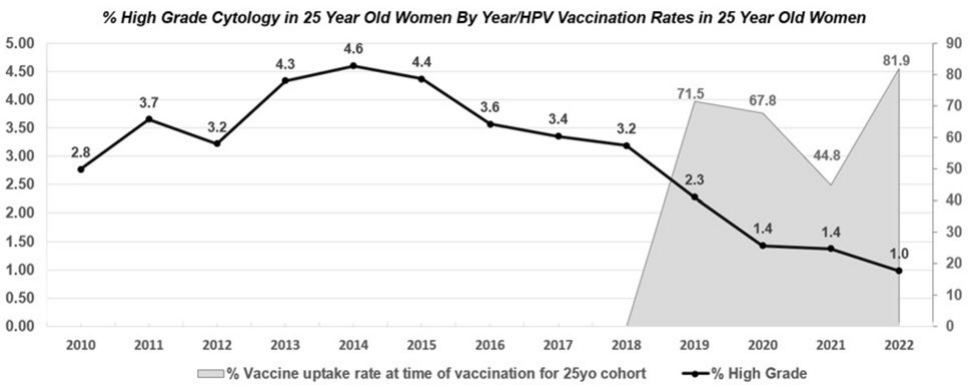
Percentage of high-grade cytology by year and HPV vaccination rates in 25-year-old women

In the period before this cohort joined the cervical screening programme, the results show an increase in HG cytology in 25-year-olds from 2.7 to 4.6% in 2010–2014, reflective of the initial increase expected of a new screening programme, followed by a reduction from 4.4 to 2.3% in 2015–2019 (Fig. 1). The reduction becomes steeper as further vaccinated cohorts become eligible in 2019–2022, reducing from 2.3 to 1.0%. The fluctuation in vaccination rates (44.8% in 2013/2014 and 81.9% in 2014/2015) may reflect fewer eligible girls in 2012/14 as most girls had been vaccinated in the previous year and may have not taken transition year in school.

The rate of HG cytology in 25-year-olds for the period 2015–2018 was 3.7% of all cytology tests taken in this age group. For the corresponding period from 2019 to 2022, the average percentage of HG cytology in 25-year-olds was 1.5%, representing a significant reduction in HG cytology proportions between the two periods (*p* < 0.001).

## Discussion

This study gives an early indication outside the randomised controlled setting of the impact of HPV vaccination on high-grade cytology and adds to the growing international evidence that a combination of screening and HPV vaccination can lead to cervical cancer elimination. A Cochrane Review concluded that there is high-certainty evidence that HPV vaccines protect against cervical precancer in adolescent girls and young women aged 15 to 26. The effect is higher for lesions associated with HPV16/18 than for lesions associated with other HPV types. The effect is greater in those who are negative for hrHPV or HPV16/18 DNA at enrolment than those unselected for HPV DNA status [12].

There have been developments in vaccination since that Cochrane Review, including the introduction of Gardasil 9 and gender-neutral vaccination in Ireland in September 2019. Gardasil 9 protects against the 4 HPV subtypes found in Gardasil 4 and further 5 subtypes responsible in total for 90% of cervical cancer [9]. The introduction of HPV vaccination in boys will help to provide herd immunity to unvaccinated girls [9]. As initial vaccination rates of catch-up groups were low, it is expected that as the cohorts with higher vaccination coverage become eligible for screening, the protective effect of HPV vaccination will have a more robust effect on HG disease. HPV vaccination in adolescence is currently associated with up to a 90% reduction in cervical cancer and its full population-level benefits will only materialise when vaccinated adolescents reach mid to late adulthood [8]. As these positive developments materialise, the potential for increased screening intervals, a change in the number of lifetime tests for women vaccinated with Gardasil 9, and the prescribed maximum age for vaccination will grow [9].

The main limitation of this study is the unavailability of data on individual participant vaccination status; thus the vaccination data and screening results were not linked at an individual level. Therefore, the results of the study were inferential as an individual identifier for health data remains confined to pilot sites in Ireland. A Swedish population-level study [13] recently showed the benefits of a linked individual identifier (unique personal identity number) that allowed the retrieval of information on immigration, emigration, death, and other personal characteristics facilitating accurate extrapolation of the efficacy, effectiveness, and herd immunity impact of vaccination on precancerous lesions and invasive cervical cancer. Current work is in progress in Ireland to merge individual-level vaccination data from the National Immunisation Office (NIO) with the Irish cervical screening register, which will allow a more detailed assessment of the impact of vaccination on cervical disease in the future. The decline in high-grade disease which preceded the introduction of free HPV vaccination is likely to represent uptake of vaccination outside of the national programme, where individuals paid for vaccines privately. However, this remains speculative, as vaccinations administered in the private sector in Ireland are not recorded by the NIO. The strengths of the study include a national cervical screening programme, a national HPV vaccination programme, and good quality data in each setting.

## Conclusion

This study provides early evidence of the potential impact of HPV vaccination on cervical disease in the Republic of Ireland (which supports the HPV vaccination, cervical screening, and cancer treatment approach of the WHO Strategy for Cervical Cancer Elimination). Despite lower vaccination uptake in the initial catch-up group, we are seeing early signs of the positive protective effect of vaccination in women at the time of their first cervical screening test. As the cohorts with higher vaccination coverage become eligible for screening, we can expect a greater impact on HG disease. Plans to incorporate individual-level vaccination status for women on the cervical screening register will allow a more detailed assessment of the impact of HPV vaccination in the Irish population. Future changes to cervical screening will need to incorporate vaccination data into algorithms for screening intervals and it is likely that individualised risk profiling will play a greater role; current research is addressing what that might look like in a population screening programme [14].
